# Supplementary material for: Skin and fur bacterial diversity and community structure on American southwestern bats: effects of habitat, geography and bat traits
Source: PeerJ. 2017 Oct 27;5:e3944. doi: 10.7717/peerj.3944 (PMC5661439; doi:10.7717/peerj.3944)
Supplement: Supplemental Information 2 — Most abundant phyla found on bats in the study separated by bat species. I is the most abundant phyla for that species, II the second most abundant phyla and III the third most abundant phyla based on relative abundance. [file peerj-05-3944-s002.pdf]

Supplemental table 2. Most abundant phyla found on bats in the study separated by bat species. I is the most abundant phyla for that species, II the second most abundant phyla and III the third most abundant phyla based on relative abundance.

**Bat species**	**I**	**II**	**III**
A. pallidus	Actinobacteria	Alphaproteobacteria	Cyanobacteria
C. townsendii	Actinobacteria	Alphaproteobacteria	Acidobacteria
E. fuscus	Actinobacteria	Firmicutes	Alphaproteobacteria
L. noctivagans	Actinobacteria	Alphaproteobacteria	Betaproteobacteria
M. californicus	Actinobacteria	Alphaproteobacteria	Cyanobacteria/ Acidobacteria
M. ciliolabrum	Actinobacteria	Alphaproteobacteria	Chloroflexi
M. evotis	Actinobacteria	Alphaproteobacteria	Gammaproteobacteria
M. occultus	Actinobacteria	Alphaproteobacteria	Firmicutes
M. thysanodes	Actinobacteria	Firmicutes	Gammaproteobacteria
M. velifer	Actinobacteria	Gammaproteobacteria	Bacteroidetes
M. volans	Actinobacteria	Gammaproteobacteria	Alphaproteobacteria
P. hesperus	Actinobacteria	Alphaproteobacteria	Cyanobacteria
T. brasiliensis	Firmicutes	Actinobacteria	Alphaproteobacteria
