# Supplementary figures and images for: Skin and fur bacterial diversity and community structure on American southwestern bats: effects of habitat, geography and bat traits
Source: PeerJ. 2017 Oct 27;5:e3944. doi: 10.7717/peerj.3944 (PMC5661439; doi:10.7717/peerj.3944)

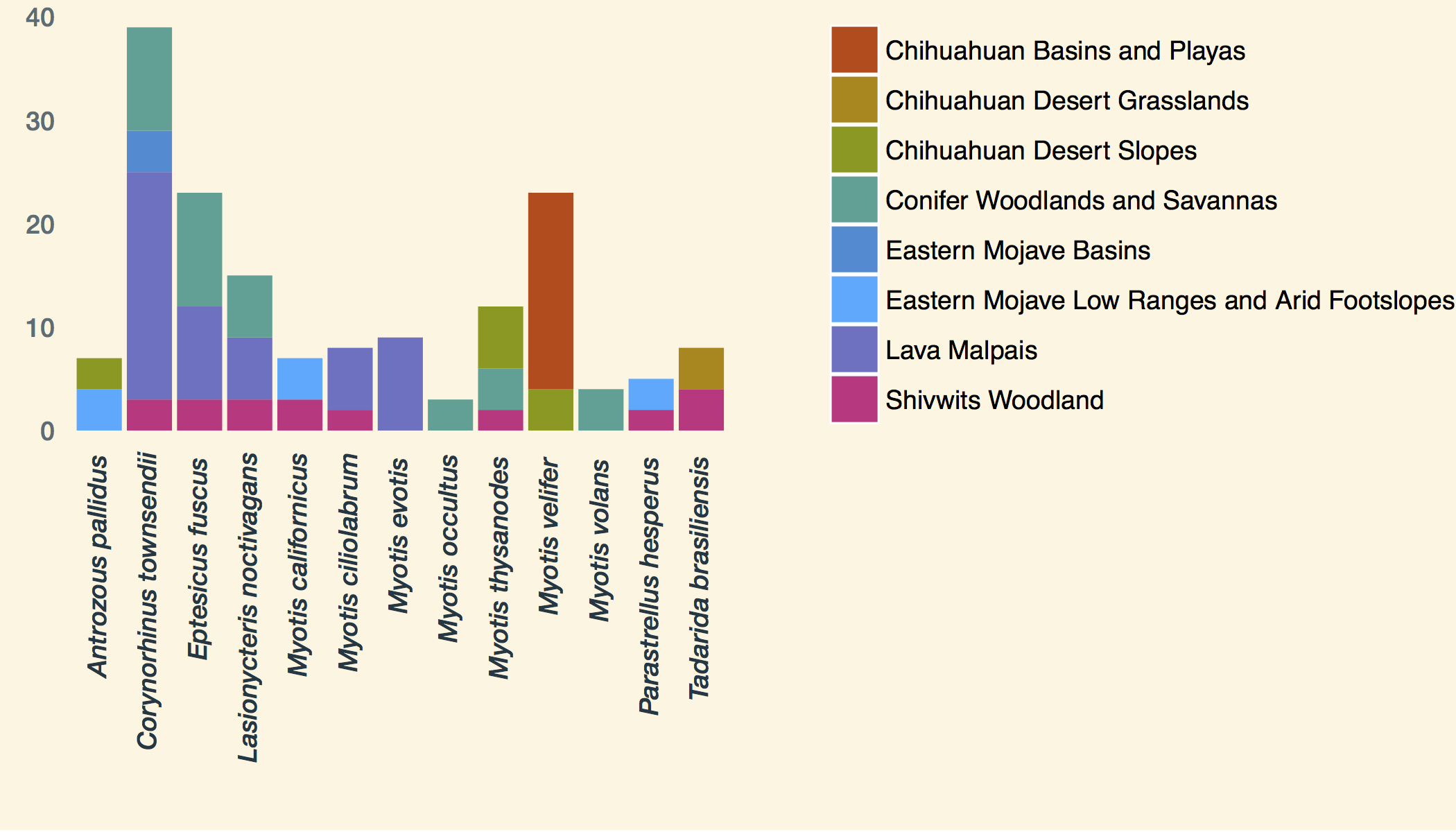

Supplement: Supplemental Information 3 — Counts of bats sampled colored by the ecoregion in which the bat was caught. [file peerj-05-3944-s003.png]

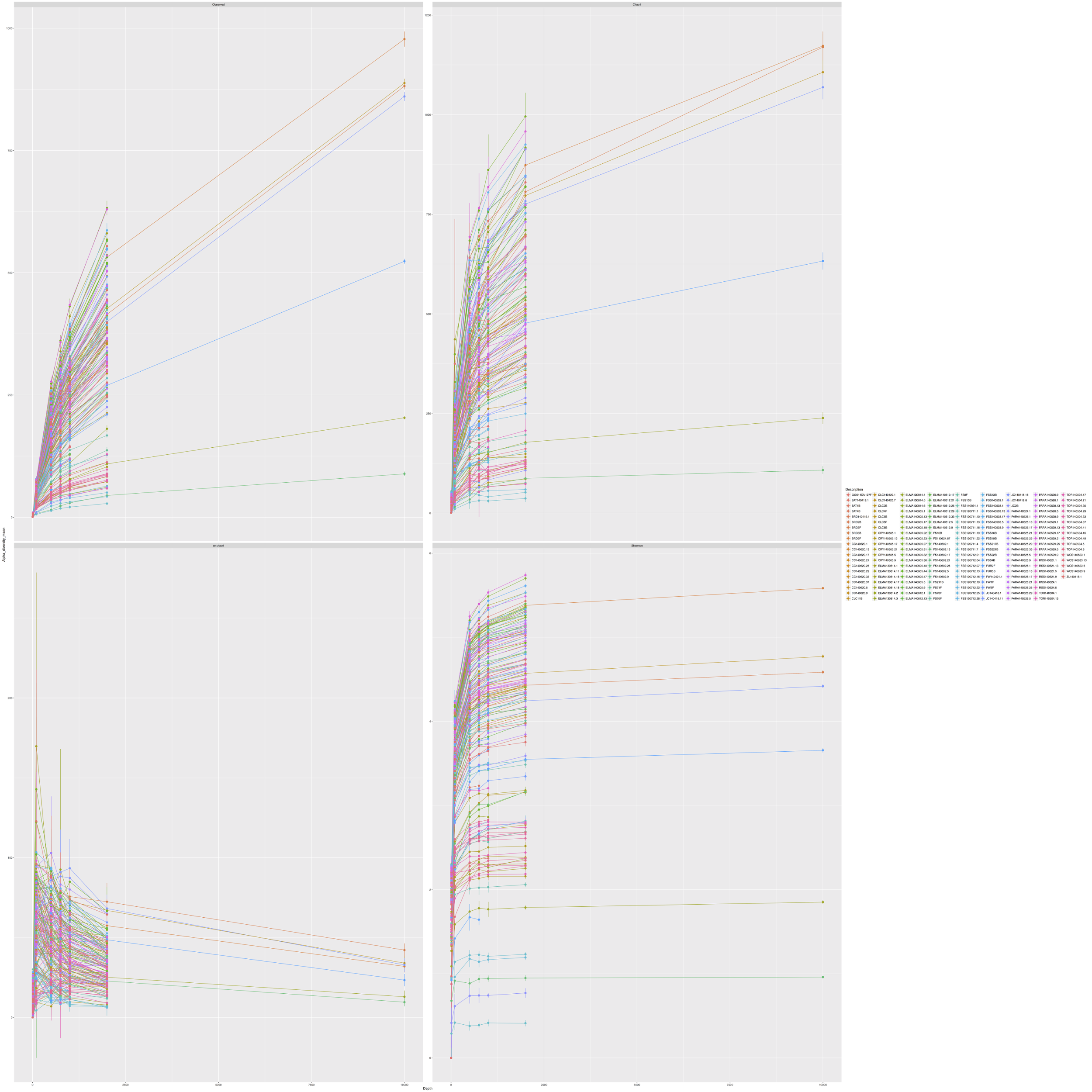

Supplement: Supplemental Information 4 — Rarefaction curves showing sampling error across different alpha diversity indices. [file peerj-05-3944-s004.png]

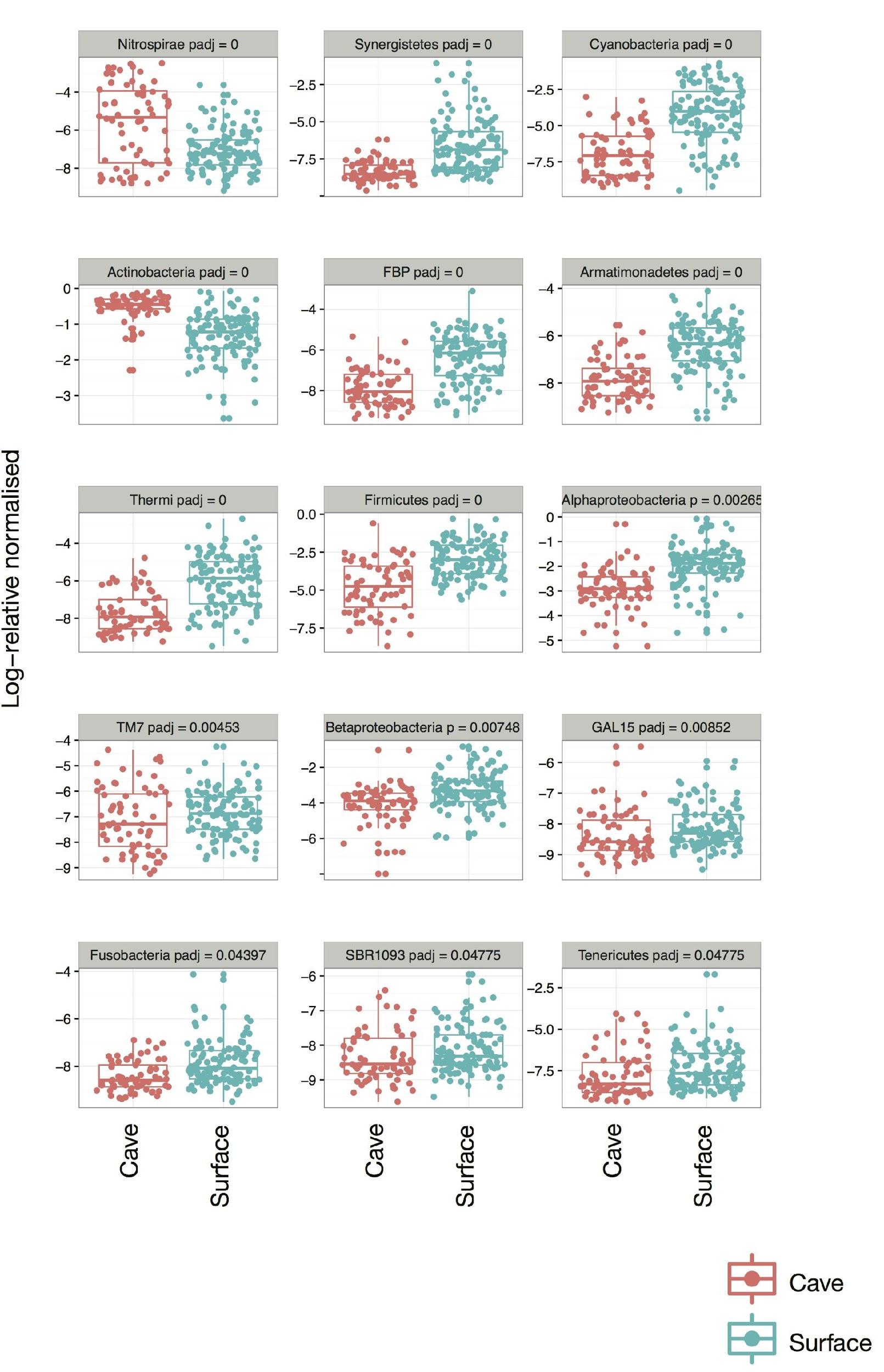

Supplement: Supplemental Information 5 — Differential abundance of OTUs between cave-caught and surface-netted bats using DESeq2. Two OTUs at the phylum level were higher in cave-caught bats: Actinobacteria and Nitrospira. [file peerj-05-3944-s005.jpeg]
